# Incidence of active tuberculosis among people living with HIV receiving long‐term antiretroviral therapy in high TB/HIV burden settings in Thailand: implication for tuberculosis preventive therapy

**DOI:** 10.1002/jia2.25900

**Published:** 2022-04-05

**Authors:** Gompol Suwanpimolkul, Sivaporn Gatechompol, Kamon Kawkitinarong, Thornthun Ueaphongsukkit, Jiratchaya Sophonphan, Nirada Siriyakorn, Supunnee Jirajariyavej, Suwimon Khusuwan, Palakorn Panarat, Surat Wannalerdsakun, Natcha Saetiew, Sunee Chayangsu, Sirichai Wiwatrojanagul, Preudtipong Noopetch, Praniti Danpornprasert, Sripetcharat Mekviwattanawong, Chris Fujitnirun, Cheewanan Lertpiriyasuwat, Win Min Han, Stephen J. Kerr, Kiat Ruxrungtham, Anchalee Avihingsanon, and the HIV‐TB study team

**Affiliations:** ^1^ Division of Infectious Disease Department of Medicine Faculty of Medicine Chulalongkorn University King Chulalongkorn Memorial Hospital Bangkok Thailand; ^2^ Centre of Excellence in Tuberculosis Division of Pulmonary Disease Department of Medicine Faculty of Medicine Chulalongkorn University Bangkok Thailand; ^3^ Emerging Infectious Diseases Clinical Center Bangkok Thailand; ^4^ HIV‐NAT Thai Red Cross AIDS Research Centre Bangkok Thailand; ^5^ Division of Pulmonary Disease Department of Medicine Faculty of Medicine Chulalongkorn University Bangkok Thailand; ^6^ Infectious Disease Unit Medicine Department Rajavithi Hospital Bangkok Thailand; ^7^ Medicine Department Taksin Hospital Bangkok Thailand; ^8^ Medicine Department Chiangrai Prachanukroh Hospital Chiangrai Thailand; ^9^ Medicine Department Queen Savang Vadhana Memorial Hospital Chonburi Thailand; ^10^ Division of Infectious Disease Department of Medicine Naresuan University Hospital Phitsanulok Phitsanulok Thailand; ^11^ Medicine Department Sisaket Hospital Sisaket Thailand; ^12^ Medicine Department Surin Hospital Surin Thailand; ^13^ Medicine Department Maharat Nakhon Ratchasima Hospital Nakhon Ratchasima Thailand; ^14^ Medicine Department Hatyai Hospital Hatyai Thailand; ^15^ Medicine Department Klang Hospital Bangkok Thailand; ^16^ Medicine Department Pranangklao Hospital Nonthaburi Thailand; ^17^ Medicine Department Bhumibol Adulyadej Hospital Bangkok Thailand; ^18^ Division of AIDS and STIs Department of Disease Control Ministry of Public Health Nonthaburi Thailand; ^19^ The Kirby Institute University of New South Wales Sydney New South Wales Australia; ^20^ Biostatistics Excellence Centre Research Affairs Faculty of Medicine Chulalongkorn University Bangkok Thailand

**Keywords:** antiretroviral therapy, Asia, HIV, incident tuberculosis, latent TB infection, Thailand

## Abstract

**Introduction:**

Among high tuberculosis (TB) and HIV burden countries in Asia, tuberculosis preventive therapy (TPT) in people living with HIV (PLWH) has been underutilized despite its proven benefits independent of antiretroviral therapy (ART). Therefore, we determined the incidence of active TB and mortality among 9179 adult PLWH who attended and received ART from 15 tertiary care hospitals across Thailand.

**Methods:**

A retrospective study was conducted in 2018 using follow‐up data from 1999 to 2018. The primary endpoint was incident TB disease after ART initiation. Factors associated with TB incidence were analysed using competing risk regression. The Kaplan–Meier method was used to estimate mortality after ART initiation.

**Results:**

During a median of 5.1 years of ART (IQR 2.2–9.5 years), 442 (4.8%) PLWH developed TB (TB/HIV), giving an overall incidence of 750 (95% CI 683–823) per 100,000 persons‐year of follow up (PYFU). In multivariate analysis, lower CD4 at ART initiation (≤100 cells/mm^3^, adjusted sub‐distribution hazard ratio [aSHR]: 2.08, 95% CI, 1.47–2.92; 101–200 cells/mm^3^, aSHR: 2.21, 95% CI, 1.54–3.16; 201–350 cells/mm^3^, aSHR: 1.59, 95% CI, 1.11–2.28 vs. >350 cells/mm^3^), male sex (aSHR: 1.40, 95% CI, 1.11–1.78), lower body weight (<50 kg, aSHR: 1.52, 95% CI, 1.17–1.95) and prior TB event (aSHR: 3.50, 95% CI, 2.72–4.52) were associated with TB incidence. PLWH with HIV RNA ≥50 copies/ml had 5–9 times higher risk of active TB disease higher than those with HIV RNA <50 copies/ml at the same CD4 level. The risk for developing TB was remarkably high during the initial period of ART (175,511 per 100,000 PYFU at<3 months) and was comparable to the general population after 10 years of ART (151 per 100,000 PYFU). TB/HIV had higher mortality (10% vs. 5%) and poorer HIV treatment outcomes: HIV RNA <50 copies/ml (63.8% vs. 82.8%), CD4 cells count (317 vs. 508 cells/mm^3^) at the most recent visit.

**Conclusions:**

In this high TB burden country, TB incidence was remarkably high during the first few years after ART initiation and thereafter decreased significantly. Rapid ART initiation and appropriate TPT can be potential key interventions to tackle the TB epidemic and reduce mortality among PLWH in TB/HIV high burden settings.

## 1 INTRODUCTION

Despite the availability of effective antiretroviral therapy (ART), tuberculosis (TB) remains the leading cause of morbidity and mortality among people living with human immunodeficiency virus (PLWH). The risk of developing active TB disease is estimated to be 20 times greater in PLWH than those without HIV infection [1]. In 2019, an estimated 820,000 PLWH worldwide had active TB and 208,000 PLWH died from TB [1]. Thailand had an estimated TB incidence of 150 cases per 100,000 persons in the general population in 2019, which decreased slowly from 172 cases per 100,000 persons in 2012. In 2021, the World health Organization (WHO) announced that Thailand retained its position as a high TB burden and high TB/HIV burden country. In 2019, over 13% of TB patients had HIV infection, and there were 10,000 TB cases and 1900 TB‐related deaths among PLWH [1]. Although ART results in a time‐ and CD4‐dependent reduction in the risk of developing active TB by 65%, [2] and Thailand has provided free ART for all PLWH regardless of CD4 cell count since 2014, only 82% of individuals with TB/HIV co‐infection were on ART in 2019 [1].

Addition of TB preventive therapy (TPT) to ART has been shown to further reduce TB incidence by 30% [3, 4, 5] and mortality rate by 35–50% [6, 7], and it is recommended by WHO. Almost half the global TB burden is from Asia and the pacific; however, PLWH who receive TPT in Asia remains relatively low compared to Africa [1]. TPT has not been successfully implemented throughout Thailand, and only 0.4% of PLWH received TPT in 2019 [1]. TB incidence is highest in the first 3 months of after initiating ART, and the risk of TB declines proportionally as CD4 cell counts increase [2, 8]. Nevertheless, data from high TB burden countries in Africa have shown the risk of developing active TB after ART initiation with high CD4 counts (>700 cells/mm^3^) is four times higher compared to those without HIV infection [9].

To develop evidence‐based strategies for preventing active TB disease in PLWH on ART, it is important to have more data on the incidence of active TB and the risk factors for developing TB post‐ART initiation, especially in PLWH with long‐term HIV viral suppression. Unfortunately, there are limited data from high TB burden countries in the Asia‐Pacific region, particularly among PLWH who have been taking on ART for more than 5 years. These data could contribute a better understanding of how implementation of additional preventive strategies, such as TPT during the first few years of ART initiation, could improve health outcomes. We, therefore, investigated the incidence of and risk factors for incident TB, using the HIV cohorts from 15 hospitals across Thailand. We additionally assessed mortality associated with TB/HIV.

## 2 METHODS

### 2.1 Study participants and design

Data were retrospectively collected and analysed from 15 HIV clinics across Thailand with integrated HIV and TB services. The inclusion criteria were PLWH aged ≥18 years who initiated ART from 1999 to 2017. According to the Thai National HIV program guidelines, PLWH were followed every 3–6 months; CD4 cell counts were assessed at baseline then every 6 months. HIV RNA was performed at 6 months after ART and then every 6 months until HIV RNA <50 copies/ml, and annually thereafter. All PLWH were screened for active TB prior to starting ART, which included symptom screening, physical examination and chest radiography (CXR). During the follow‐up period, participants with symptoms and/or signs of pulmonary or extrapulmonary TB were assessed as per Thailand's National TB treatment guidelines, which include physical examination, CXR, sputum smear microscopy, culture for *M. tuberculosis* and/or GeneXpert MTB/RIF (from 2012 onwards) as indicated.

Relevant covariates were collected retrospectively by reviewing the patient's medical record, including HIV transmission mode, history of AIDS defining illnesses, CD4 cell count at ART initiation, type and duration of ART regimens and type of TB (defined as pulmonary, extrapulmonary or disseminated TB). All TB cases included in the study were clinically diagnosed or bacteriologically confirmed TB, retrieved from the medical records. This study was approved by the Ethics Committee of the Faculty of Medicine, Chulalongkorn University, Bangkok, Thailand and local ethics committees of the other 14 hospitals. All cohort participants provided written inform consent.

### 2.2 Statistical analysis

Demographic, clinical and laboratory parameters were described for the cohort overall, and for groups of PLWH stratified by TB incidence. Differences in continuous and categorical covariates between incident TB (TB/HIV) and non‐TB groups were assessed using a Wilcoxon rank sum test and a Chi‐square test, respectively. All *p*‐values reported are two‐sided, and statistical significance was defined as *p*<0.05. Competing risk regression [10] was used to calculate the cumulative TB incidence function and derive sub‐distribution hazard ratios (SHRs) for covariates potentially associated with this outcome. Deaths, transfers and losses‐to‐follow‐up were considered competing risks. The Kaplan–Meier method was used to estimate mortality incidence in the PLWH with TB and without TB groups. Lost to follow‐up was defined as not attending clinic for 12 months from their previous appointment. Follow‐up continued until 31 July 2018, and patients without an outcome in the relevant models were censored on this date; in the mortality analysis, patients were censored on the last date they were known to be alive and in care. TB incidence rates were calculated based on total person follow‐up to facilitate comparison with other studies. The following baseline covariates were assessed for their association with incident TB: age, sex, CD4 cell count, history of TB before ART initiation, body weight and during follow‐up chronic kidney disease (CKD‐EPI <60 ml/min/1.73 m^2^), diabetes mellitus and chronic liver disease (chronic hepatitis B and chronic hepatitis C), CD4 cell counts and HIV RNA <50 copies/ml (yes or no). Multivariate models were developed by adjusting for the covariates with *p*<0.1 in univariate models, and backward stepwise methods were used to select a final model. Stata version 15.1 (Stata Corp., College Station, TX) was used for analysis.

## 3 RESULTS

**Figure 1:**
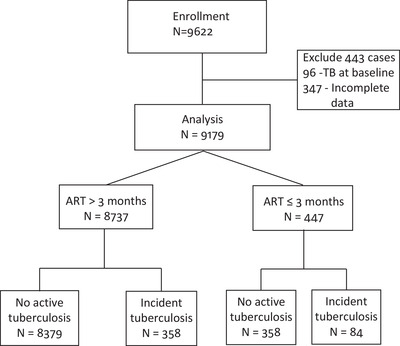
Study schema.

Overall, medical records of 9622 PLWH were reviewed and 443 PLWH were excluded due to incomplete data or because they had TB at baseline, leaving 9179 participants for analysis. The study schema is depicted in Figure 1.

A total of 769 PLWH (8.6%) had TB diagnoses prior to ART initiation. Among those with history of TB, 109 (14.2%) of them further developed active TB after ART initiation (TB reinfection). During follow‐up, 486 (5.3%) died, 737 (8%) were transferred out and 962 (10.5%) were lost to follow‐up. Characteristics at ART initiation and at last follow‐up are presented in Table 1.

**Table 1:** Characteristics of participants among PLWH with TB and without TB

	Total	Without TB	With TB	
	(*N* = 9179)	(*N* = 8737)	(*N* = 442)	*p*‐Value
At time of antiretroviral therapy initiation				
Age (years)	34.5 (28–41.7)	34.5 (28–41.7)	34.1 (28.2–41.4)	0.87
Male, *N* (%)	5609 (61.1)	5319 (60.9)	290 (65.6)	0.04
CD4 cell count, cells/mm^3^	227 (81–384)	232 (84–388)	141 (47–265)	<0.001
Body weight, kg	52.9 (48.7–60.1)	57 (50–65)	53 (48.7–60.1)	<0.001
History of prior TB, *N* (%)	769 (8.6)	660 (7.7)	109 (25.2)	<0.001
During follow‐up				
Diabetes mellitus, *N* (%)	462 (5)	441 (5.0)	21 (4.8)	0.78
Chronic kidney disease, *N* (%)	464 (5.1)	434 (5)	30 (6.8)	0.09
Chronic liver disease, *N* (%)	840 (9.2)	785 (9.0)	55 (12.4)	0.01
At last visit follow‐up/censoring date				
Age (years)	41.9 (33.2–49.5)	42.2 (33.3–49.7)	38.8 (31.9–45.3)	<0.001
CD4 cell count, cells/mm^3^	495 (345–670)	506 (354–677)	317 (141–483)	<0.001
CD4 cell count group, *N* (%)				<0.001
• <200	821 (8.9)	682 (7.8)	139 (31.5)	
• 201–350	1418 (15.5)	1327 (15.2)	91 (20.6)	
• 351–500	2167 (23.6)	2063 (23.6)	104 (23.5)	
• >500	4280 (46.6)	4183 (47.9)	97 (22)	
• Unknown	493 (5.4)	482 (5.5)	11 (2.5)	
HIV‐RNA <50 copies/ml, *N* (%)	7513 (81.9)	7231 (82.8)	282 (63.8)	<0.001
Duration of ART (years)	5.1 (2.2–9.5)	5.3 (2.4–9.6)	2.3 (0.5–5.7)	<0.001
Median (IQR)				
Death, *N* (%)	486 (5.3)	442 (5.1)	44 (10)	<0.001

Note: Data presented as median (IQR) and *N* (%), Wilcoxon rank sum test was used to compare continuous distributions and a Chi‐square test was used to compare proportion.

Median baseline CD4 count was 227 cells/mm^3^; median age at ART initiation was 34.5 years; and 61% were male. At ART initiation, the incident TB group had lower CD4 cell counts, lower body weight and higher proportion of prior TB events. At last follow‐up, individuals with incident TB were significantly younger, had lower CD4 cell counts, a lower proportion with HIV RNA <50 copies/ml, shorter ART duration and higher death rates.

### 3.1 TB incidence over time

**Figure 2:**
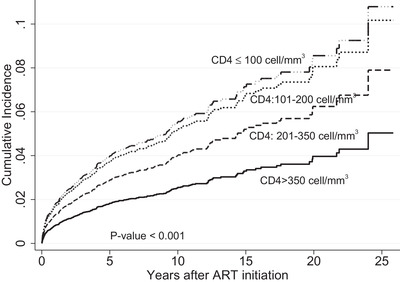
Cumulative TB incidence rate stratified by CD4 cell count at ART initiation.

During a median of 5.1 years of ART, 442 PLWH developed incident TB, resulting in an overall incidence rate of 750 (95% CI 683–823) per 100,000 persons year follow up (PYFU). Almost 19% of incident TB cases occurred in the first 3 months of ART initiation. Table S1 reports the incidence rate per 100,000 PYFU at each year after starting ART. TB incidence was much higher during the first year of ART, especially during the first 3 months. The TB incidence declined over time, with an incidence of 175,511 per 100,000 PYFU during first 3 months compared to an incidence of 151 per 100,000 PYFU after ≥10 years on ART. TB incidence was highest among PLWH with CD4 <100 cells/mm^3^ (1153 per 100,000 PYFU) and 436 per 100,000 PYFU for those with CD4 >500 cells/mm^3^ (Table S2). Of note, those with unknown baseline CD4 cell counts had an incident TB rate of 960 per 100,000 PYFU. Figure 2 shows the cumulative incidence of TB at different baseline CD4; the lower CD4 cell counts, the higher active TB incidence.

At the time of TB diagnosis (9% missing CD4 data), the median CD4 count was 179 (IQR 69–392) cells/mm^3^. Nearly, 20%, 12%, 18%, 15%, 14% and 13% of TB events occurred at the time of CD4 <50, 51–100, 101–200, 201–350, 351–500 and >500 cells/mm^3^, respectively. Importantly, only 183 (41%) participants had HIV RNA < 50 copies/ml at TB diagnosis. The median time from ART initiation to TB diagnosis was 2.3 (IQR 0.5–5.7) years. Almost 75% had pulmonary TB, and 17% and 8% had extrapulmonary TB and disseminated TB, respectvely. Table S3 shows the characteristics of incident TB among those with and without HIV supression (<50 copies/ml). Of 305 TB/HIV co‐infected individuals with HIV RNA available at time of TB diagnosis, 183 (41%) had HIV RNA <50 copies/ml. Those with HIV RNA >50 copies/ml had lower CD4 cell counts (89 vs. 337) cells/mm^3^, had higher percentage of CD4 <100 cells/mm^3^ (48.7% vs. 30.1%) and had shorter duration of ART (1.7 vs. 3.1 years). For those with HIV RNA <50 copies/ml, the incidence rate of TB was the highest in those with CD4 ≤100 cells/mm^3^ (805 per 100,000 PYFU) (Table S4). Similarly, among those with HIV RNA ≥50 copies/ml, the incidence rate of TB was higher in those with CD4 ≤100 cells/mm^3^ (5100 per 100,000 PYFU compared to 1505 per 100,000 PYFU in >500 cells/mm^3^). In addition, the TB incidence was higher in those with HIV RNA ≥50 copies/ml and CD4 >500 cells/mm^3^ than those with HIV RNA <50 copies/ml and CD4 >500 cells/mm^3^ (1505 vs. 330 per 100,000 PYFU).

### 3.2 Risk factors associated with TB incidence after ART initiation

Of 442 incident TB events after ART initiation, 84 (19.0%) occurred within 3 months; 32 (7.2%) at 3–6 months after ART initiation; 39(8.8%) at 6–12 months after ART initiation; and 267 (60.4%) after 12 months on ART (Table 2).

**Table 2:** Risk factors associated with TB incidence after ART initiation

	Univariate		Multivariate	
	SHR (95% CI)	*p*‐value	aSHR (95% CI)	*p*‐value
At ART initiation				
Age (years)	0.94 (0.78–1.13)	0.55		
Male	1.35 (1.11–1.65)	<0.001	1.40 (1.11–1.78)	0.003
CD4 cell count, cells/mm^3^				
• ≤100	2.79 (2.10–3.72)	<0.001	2.08 (1.47–2.92)	<0.001
• 101–200	2.54 (1.86–3.46)	<0.001	2.21 (1.54–3.16)	<0.001
• 201–350	1.48 (1.07–2.04)	0.02	1.59 (1.11–2.28)	0.01
• >350	Ref		Ref	
Weight <50 kg	1.38 (1.10–1.74)	0.005	1.52 (1.17–1.95)	0.001
Prior TB event	3.67 (2.97–3.58)	<0.001	3.50 (2.72–4.52)	<0.001
During follow‐up				
Diabetes mellitus	0.71 (0.45–1.10)	0.13		
Chronic kidney disease	0.87 (0.59–1.27)	0.47		
Chronic liver disease	1.39 (1.05–1.84)	0.02	0.95 (0.67–1.35)	0.79

In multivariate analysis, male sex: aSHR 1.40 (95% CI 1.11–1.78), lower baseline CD4 (<100 cells/mm^3^, aSHR: 2.08, 95% CI 1.47–2.92, 101–200 cells/mm^3^, aSHR: 2.21, 95% CI 1.54–3.16), 201–350 cells/mm^3^, aSHR: 1.59, 95% CI 1.11–2.28 vs. >350 cells/mm^3^), lower baseline body weight (<50 kg, aSHR: 1.52, 95% CI 1.17–1.95) and prior TB event (aSHR: 3.50, 95% CI 2.72–4.52) were significantly associated with TB incidence. In a separate analysis when both age and diabetes mellitus adjusted for in the multivariate model, similar results were obtained.

### 3.3 Survival rates over time

**Figure 3:**
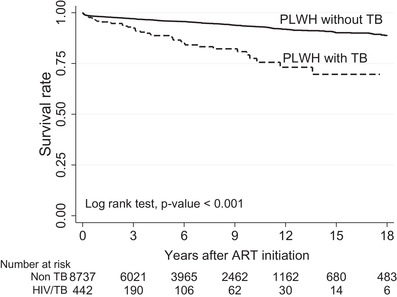
Mortality rate between PLWH with and without incident TB.

In 8737 PLWH without incident TB, 442 (5%) died. Of 442 PLWH with incident TB, 44 (10%) died. The median time to death from ART initiation was 2.8 (IQR 0.7–5.6) years. Overall survival rate at the end of follow‐up was 77.8% for PLWH without incident TB and 69.6% for those with incident TB (*p*‐value <0.001) (Figure 3).

## 4 DISCUSSION

In this cohort of PLWH from 15 tertiary hospitals across Thailand with a median duration of 5 years on ART, the overall TB incidence rate was 750 per 100,000 PYFU compared with that in the general Thai population of 150 per 100,000 PYFU in 2019 [1]. This study is important because the data were obtained from a high TB and HIV burden country in Asia with a longer follow‐up duration. Most previously published data were from African countries or from low to intermediate TB burden countries [9, 10, 11, 12, 13, 14, 15, 16, 17]. Although TB screening was performed prior to ART initiation and at each visit, the incidence of TB was significantly higher during the first year of ART initiation, especially during the first 3 months. Our findings are consistent with other previous studies from high TB burden that TB incidence was the highest within the first few months of ART initiation, which may partly be due to unmasking of TB, which is also known as immune recovery syndrome [18, 19]. However, the high TB incidence rates in the initial period of ART initiation in our study may be attributed to inadequate TB screening, and this also highlights the importance of TB screening at ART initiation, especially among those starting ART with very low CD4 levels.

Previous studies from Europe and the United States (low TB burden countries) reported the TB incidence to be 1300 cases per 100,000 PYFU within 90 days of starting ART compared to countries in Africa, where the TB incidence rates ranged from 900 to 7900 per 100,000 PYFU [10, 11, 12, 13, 14, 15, 16, 17]. Our findings demonstrated that TB incidence was 17,500 per 100,000 PYFU within 90 days of starting ART. This suggests that the TB incidence in PLWH adults also depends on the background prevalence of HIV and TB in that country.

As expected, we found high incidence of TB in PLWH with lower baseline CD4 at ART initiation. The lower CD4, the higher the number of incident TB cases. In those with CD4 <100 cells/mm^3^, the TB incidence was 1153 per 100,000 FYFU compared to 360 per 100,000 PYFU among those with CD4 350–500 cells/mm^3^. In multivariate analysis, low CD4 count at ART initiation significantly increased the risk of developing TB. TB incidence was also notably high for PLWH with good immune status (CD4> 500 cells/mm^3^) – approximately three times higher than the general population. Moreover, it is likely that active TB was also driven by HIV viremia. Our findings found that those with HIV RNA >50 copies/ml had higher risk of developing active TB disease than those with HIV RNA <50 copies/ml at the same CD4 levels. Despite having high CD4 count, the risk of active TB remained high for those with HIV viremia. Of note, HIV viremic individuals with incident TB in our study had much shorter ART duration than those with viral suppression. This finding corroborates the data from the START study and TEMPRANO study [7, 20], and it supports the rapid ART strategy to facilitate HIV viral suppression as soon as possible, aiming to reduce death and TB reactivation risks. Therefore, rapid ART strategy should be implemented regardless of CD4 cell counts. Recent findings from the HPTN 071 (PopART) trial also confirmed that PLWH who have started ART at CD4 count higher than 500 cells/mm^3^ in high TB burden setting of South Africa had no incident TB cases observed [21]. The sharp decrease in TB incidence after first year of ART initiation in our cohort is partly due to the result of immune restoration after HIV RNA was suppressed. In addition, the implementation of “treat‐all” policy irrespective of CD4 count and wider access to ART in the region at recent years would also impact the TB incidence among PLWH population in Thailand.

Apart from higher risk of developing TB with low CD4, we found that male and PLWH with low body weight were also associated with increased risk of incident TB. The association between body mass index (BMI) and TB incidence has been reported in diverse populations with and without HIV infection. The study from Tanzania also found that underweight PLWH had a two‐fold increased risk of TB, while in those who were overweight and obese, the risk was reduced by 36% and 45%, respectively [15]. The negative effect of low body weight/BMI on TB might be due to altered immunity. There is also evidence that individuals with higher visceral fat (BMI 18.5–30 kg/m^2^) release higher amount of tumour necrotic factor and other pro‐inflammatory markers that are critical immune mediator in protection against TB reactivation [22, 23]. A systemic review and meta‐analysis reported that active TB is significantly higher among men than women in low‐ and middle‐income countries and men are disadvantaged in seeking and/or accessing TB care in many settings [24]. Our findings of PLWH with prior TB had 3.5‐fold increased risk of developing TB, supporting the WHO guideline on the use of TPT. According to the recent WHO guideline 2020, prior TB is included among the recommendations of TPT use [25]. Since the association of prior TB and incident TB among our PLWH was significant, especially for those with low CD4 and detectable HIV RNA, TPT should be implemented in this population, particularly for those with previously treated TB for more than 2 years.

Although ART reduces the risk of active TB by 67% [8], the risk of TB declines when the CD4 cell count increases after ART initiation [2]. However, ART alone might not be sufficient to prevent TB among PLWH. Data from Cape Town, South Africa, a high TB burden country, showed that the risk of active TB after ART initiation among participants with CD4 >700 cells/mm^3^ remained four times higher than that in HIV‐negative population [9]. Our findings also showed that PLWH with VL suppression and CD4 >500 cells/mm^3^ the risk of active TB remained two times higher than the general population, although the overall incidence declined significantly and was comparable to the general population after 10 years of ART. It is a challenge to prevent TB in participants who have just started ART because of the time taken for immune reconstitution. Therefore, TPT is an important strategy to prevent active TB while the patients’ immune system is recovering during initial period of ART initiation, especially in PLWH living in high TB burden settings.

Unfortunately, the uptake of TPT is relatively low in many Asian countries even though Asia and pacific have the highest TB burden. Although studies are lacking in the Asia‐Pacific region on why there is low TPT uptake, several studies have reported the providers’ barriers in initiating TPT, including lack of training and their perceptions on its efficacy or clarity of TPT guidelines [26, 27, 28]. Thailand is among the top 14 high burden (highest TB and TB/HIV) countries [1]. If TB reservoirs are not eliminated, then it is difficult to prevent active TB disease development among PLWH. This in turn will make it more difficult to eliminate TB in high burden settings. Moreover, active TB after ART initiation was also associated with a higher mortality rate. Our findings showed that PLWH with incident TB had higher mortality rates than those without TB, as 10% of them died at a median time after ART initiation of 2.8 years. Importantly, overall survival rate at the end of follow‐up was 77.8% for PLWH without TB and it was only 69.6% for those with TB. In addition, TB incidence is also associated with poorer HIV treatment outcomes. At the last follow‐up, PLWH with incident TB were less likely to have HIV RNA <50 copies/ml (63.8% vs. 82.8%) and their CD4 cell counts remained lower than those without TB (317 vs. 506 cells/mm^3^), also partly contributed by shorter duration of ART.

For PLWH, there is strong evidence of TPT beneficial effect in addition to ART on the reduction of TB risk, including among individuals with higher CD4 counts, especially in high TB burden country [3, 4, 5, 25, 29]. However, a recent analysis from WHO suggests that it is unlikely to achieve the TPT implementation targets in South‐East Asia Region by 2022 [30]. Without combination of early ART initiation and TPT strategy to decrease new active TB cases, TB elimination by 2030 is unlikely to accomplish. This study which included PLWH from multiple tertiary hospitals across Thailand showed that PLWH who received ART for less than 10 years, TB incidence was significantly higher than the general population. Therefore, there is an urgent need for implementing better TB screening tools and interventions, such as treating latent tuberculosis infection among PLWH. TPT should also be considered for PLWH who are newly diagnosed in high TB burden settings, particularly those who test positive for tuberculin skin test or interferon‐gamma release assays, and those who received ART therapy for less than 10 years.

A strength of this study is that it included a large number of PLWH from high TB/HIV burden in Asia with long duration of follow up after ART initiation. In addition, this study analysed the systematically collected data with most recent CD4 counts and HIV RNA levels, and the data with routine active TB screening with chest X‐ray prior to ART initiation for all. However, some limitations should be acknowledged. First, this was a retrospective data analysis, some important variables may have been missed. Second, the results of the analyses assessing the risk factors need to be interpreted with caution because several important factors related to high active TB, such as smoking, substance use, injecting drugs use and history of previous incarceration, were not included. Third, our data might not be generalizable to other settings because our participants had relatively higher baseline CD4 cell counts (227 cells/mm^3^) than the general Thai PLWH with the median baseline CD4 cells count at ART initiation of 82–192 cells/mm^3^ from the year 2009–2018 [31]. Although the National HIV program provides ART for all PLWH, almost 50% of them still presented late to care. Therefore, the incidence of active TB might be much higher than our data. Fourth, misclassification of TB outcomes cannot be totally ruled out, since PLWH may have received TB treatment and care in a clinic other than their HIV care clinic or hospital. However, this is not likely since both TB and HIV care are provided at the same hospital, according to the persons insurance scheme. In addition, PLWH were followed up every 6 months or annually, and the TB diagnoses were routinely captured in the medical records. Lastly, our data may not well represent other PLWH care in Thailand and Asia because our 15 HIV clinics included in this analysis received care from the infectious specialists in the tertiary hospitals with sufficient resources, compared to other hospital settings.

## 5 CONCLUSIONS

In this high TB burden country, TB incidence after ART initiation was remarkably high during the first few years and it was comparable to general population after 10 years of ART. The TB incidence was associated with CD4 <50 cells/mm^3^, male, low body weight and prior TB. Notably, HIV viremia had higher risk of active TB disease than those with undetectable VL at the same CD4 levels. Rapid ART initiation and intervention strategies, including TPT, can potentially be the key factors to tackle the TB epidemic and mortality rate among PLWH in TB/HIV high burden settings.

## COMPETING INTERESTS

KR has served as a consultant for Merck and Tibotec. He has had paid speaking engagements with Bristol‐Meyers Squibb, Merck, Roche, Jensen‐Cilag, GlaxoSmithKline and GPO. KR is also a Senior Researcher Scholar, Thai Research Fund (TRF); and an advisor to the National Research University Project of Commission of Higher Education (CHE) and the Ratchadaphiseksomphot Endowment Fund (HR1161A). TU is supported by Second Century Fund (C2F), Chulalongkorn University. The rest of the authors declare that they do not have any competing interests to report.

## AUTHORS’ CONTRIBUTION

GS, KK, TU, JS, SK, CL,WMH and AA conceived the study. AA, SG, GS, KK, SK, TU, NS, SJ, SK, PP, SW,NS, SC, SW, PN, PD, SM, CF and AA conducted the study. JS and SK performed the analysis and interpretation of data. GS and AA drafted the manuscript. GS, SG,, KK, TU, JS, SK, KR,WMH and AA critically revised the manuscript for intellectual content. All authors have read and approved the final manuscript.

## FUNDING

This study was supported by the Tuberculosis Research Unit (TB RU), Ratchadapiseksompotch Fund year 2021 (Chulalongkorn University code GRU 6405130010–1), the Higher Education Research Promotion and National Research University Project of Thailand, Office of the Higher Education Commission (Project code: HR 1161 A (1) and the Human Services Research Institute year 2019 in the code of 62–067 and year 2020 in the code of 63‐020 and 63‐021.

## Supporting information


**Table S1**. Incidence TB per 100,000 PYFU by duration on antiretroviral therapy.
**Table S2**. Incidence TB per 100,000/year follow‐up by CD4 at time of antiretroviral initiation.
**Table S3**. Characteristic of PLWH with TB among those with HIV‐RNA <50 copies/ml and ≥50 copies/ml at the time of TB diagnosis.
**Table S4**. TB incidence rate per 100,000 per persons year follow up stratified by CD4 count at ART initiation among PLWH with or without HIV viral suppression.

## Data Availability

The datasets generated during and/or analysed during the current study are available from the corresponding author on reasonable request.
