# Supplementary material for: Incidence of active tuberculosis among people living with HIV receiving long‐term antiretroviral therapy in high TB/HIV burden settings in Thailand: implication for tuberculosis preventive therapy
Source: J Int AIDS Soc. 2022 Apr 5;25(4):e25900. doi: 10.1002/jia2.25900 (PMC8982319; doi:10.1002/jia2.25900)
Supplement: Supplementary file 1 — Table S1. Incidence TB per 100,000 PYFU by duration on antiretroviral therapy. Table S2. Incidence TB per 100,000/year follow‐up by CD4 at time of antiretroviral initiation. Table S3. Characteristic of PLWH with TB among those with HIV‐RNA <50 copies/ml and ≥50 copies/ml at the time of TB diagnosis. Table S4. TB incidence rate per 100,000 per persons year follow up stratified by CD4 count at ART initiation among PLWH with or without HIV viral suppression. [file JIA2-25-e25900-s001.docx]

**Supplementary Table S1**. Incidence TB per 100,000 PYFU by duration on antiretroviral therapy

| **Duration on ART** | **PYFU** | **Incident TB cases** | **Incidence rate**  **(per 100,00 PYFU)** | **95% CI** | |
| --- | --- | --- | --- | --- | --- |
| ≤ 3 months | 48 | 84 | 175,511 | 141,720 | 217,359 |
| > 3 months - 6 months | 119 | 32 | 26,974 | 19,075 | 38,143 |
| > 6 months - 1 year | 310 | 39 | 12,579 | 9190 | 17,216 |
| >1 year - 2 years | 1350 | 58 | 4298 | 3323 | 5559 |
| >2 years - 3 years | 2335 | 39 | 1670 | 1220 | 2286 |
| >3 years - 4 years | 3476 | 34 | 978 | 699 | 1369 |
| >4 years - 5 years | 2527 | 30 | 1187 | 830 | 1698 |
| >5 years - 6 years | 3169 | 20 | 631 | 407 | 978 |
| >6 years - 7 years | 4039 | 18 | 446 | 281 | 707 |
| >7 years - 8 years | 3217 | 16 | 497 | 305 | 812 |
| >8 years - 9 years | 4211 | 10 | 237 | 128 | 441 |
| >9 years - 10 years | 5013 | 18 | 359 | 226 | 570 |
| >10 years | 29138 | 44 | 151 | 112 | 203 |

**Supplementary Table S2**. Incidence TB per 100,000/year follow-up by CD4 at time of antiretroviral initiation

| **CD4 at ART initiation, cells/mm^3^** | **PYFU** | **Incident TB cases** | **Incidence rate**  **(per 100,000 PYFU)** | **95% CI** | |
| --- | --- | --- | --- | --- | --- |
| ≤100 | 14,652 | 169 | 1153 | 992 | 1341 |
| 101-200 | 10,469 | 102 | 974 | 802 | 1183 |
| 201-350 | 15,579 | 88 | 565 | 458 | 696 |
| 351-500 | 9160 | 33 | 360 | 256 | 507 |
| >500 | 7113 | 31 | 436 | 306 | 620 |
| Unknown | 1978 | 19 | 960 | 613 | 1506 |
| **Total** | **58,951** | **442** | **750** | **683** | **823** |

**Supplementary Table S3**. Characteristic of PLWH with TB among those with HIV-RNA <50 copies/ml and ≥ 50 copies/ml at the time of TB diagnosis

| **At the time of TB diagnosis** | **HIV RNA ≥50 copies/mL**  **(N=122)** | **HIV RNA <50 copies/mL**  **(N=183)** | **P-value** |
| --- | --- | --- | --- |
| Age (years), median (IQR) | 32.6 (26.2-39.6) | 34.2 (28.6-40.4) | 0.13 |
| Male, N(%) | 80/122 (65.6) | 122/183 (66.7) | 0.84 |
| CD4 cell count (cells/mm^3^), median (IQR) | 89 (26.9-248) | 337 (164-488) | <0.001 |
| CD4 cell count (cells/mm^3^), N (%) |  |  | 0.007 |
| - ≤100 | 57 (48.7) | 53 (30.1) |  |
| - 101-200 | 27 (23.1) | 46 (26.1) |  |
| - 201-350 | 18 (15.4) | 49 (27.8) |  |
| - >350 | 15 (12.8) | 28 (15.9) |  |
| Body weight (kg),  median (IQR) | 53.8 (49-61) | 53 (48.7-60.9) | 0.96 |
| Duration of ART* (years), median (IQR) | 1.7 (0.5-4.5) | 3.1 (0.9-7.6) | 0.002 |

* Duration of ART was definted as the time since ART initiation to TB diagnosis

**Supplementary Table S4**. TB incidence rate per 100,000 per persons year follow up stratified by CD4 count at ART initiation among PLWH with or without HIV viral suppression

|  | **PYFU** | **Incident TB** | **Incidence rate (per 100,000 PYFU)** | **95% CI** | |
| --- | --- | --- | --- | --- | --- |
| **HIV RNA ≥50 copies/mL at last visit** |  |  |  |  | |
| CD4 cell count at ART initiation (cells/mm^3^) |  |  |  |  | |
| ≤100 | 902 | 46 | 5100 | 3820 | 6809 |
| 101-200 | 427 | 25 | 5858 | 3958 | 8670 |
| 201-350 | 754 | 22 | 2919 | 1922 | 4434 |
| 351-500 | 443 | 8 | 1804 | 902 | 3607 |
| >500 | 332 | 5 | 1505 | 627 | 3617 |
| **HIV RNA <50 copies/mL at last visit** |  |  |  |  |  |
| CD4 cell count at ART initiation (cells/mm^3^) |  |  |  |  |  |
| ≤100 | 13,038 | 105 | 805 | 665 | 975 |
| 101-200 | 9674 | 62 | 641 | 500 | 822 |
| 201-350 | 14,125 | 62 | 439 | 342 | 563 |
| 351-500 | 8330 | 19 | 228 | 145 | 358 |
| >500 | 6362 | 21 | 330 | 215 | 506 |

Abbreviations: PYFU, person-years of follow-up
